# Rehabilitation of Atrophied Maxillary Bones With Short-Splinted Implants in a Periodontitis Patient: A Six-Year Follow-Up Case Report

**DOI:** 10.7759/cureus.73514

**Published:** 2024-11-12

**Authors:** Bann AlHazmi

**Affiliations:** 1 Department of Periodontics and Community Dentistry, King Saud University, Riyadh, SAU

**Keywords:** atrophied maxillary ridge, maxillary sinus pneumatization, roxolid® dental implant, short dental implant, slactive® surface implant, splinted dental implant

## Abstract

This case report aims to report the successful use of a short-splinted implant in a patient with a history of periodontal disease. Two implants were used to rehabilitate severe atrophied alveolar ridge with fixed prosthesis. Despite the left posterior ridge being weakened by maxillary sinus pneumatization and bone remodeling after tooth extraction, no bone grafts nor sinus osteotomy procedures were needed for the rehabilitation surgery. The report examines the use of Roxolid® SLActive® surface implants (Straumann Group, Basel, Switzerland) for the restoration of missing posterior teeth in the atrophied alveolar ridge. These implants offer improved mechanical properties that enhance their durability and dependability, which increase their survival rate.

This case report demonstrates that the use of short-splinted implants can effectively reduce morbidity rates and the risk of implant failure. Additionally, the simplified rehabilitation surgery involving a short implant, when combined with proper surgical and prosthetic management, appears to be a viable treatment option that is both cost-effective and less time-consuming for rehabilitating atrophied maxillary ridges.

It is important to emphasize that rigorous periodontal maintenance, which includes regular professional follow-ups and effective oral hygiene practices, is essential for achieving optimal health of both soft and hard oral tissues around dental implants.

## Introduction

Numerous factors, including age, gender, diabetes, osteoporosis, genetic predisposition, and periodontal disease, can contribute to alveolar ridge resorption following the loss of one or more teeth [1]. As time passes after dental extractions, the amount of available bone for standard-length implant placement decreases [2]. Various surgical methods have been created to tackle this problem, such as intraoral and extraoral bone grafting, distraction osteogenesis as well as inter-positional graft, which are frequently utilized together [3,4]. However, these procedures are complex and case-specific, requiring significant time and technical skill, and may result in increased postoperative complications and overall treatment duration [4,5]. In response to these challenges, short implant placement has emerged as a viable alternative, offering reduced time, cost, morbidity, and patient discomfort compared to bone augmentation procedures for patients with inadequate residual ridges [6,7].

This case report details the rehabilitation of multiple missing teeth in the upper left posterior region using a short dental implant. Short implants have been proven to be reliable through various clinical studies [6,7]. The implants used are composed of a titanium zirconium alloy (Roxolid®; Straumann Group, Basel, Switzerland) which has a stronger tensile strength compared to pure titanium and has been shown to enhance the process of osseointegration [8]. The surface of the implants is chemically modified and treated with sandblasting, large grit, and acid etching (SLA), resulting in improved predictability for challenging treatment protocols [9].

SLActive® (Straumann Group, Basel, Switzerland) offers numerous benefits, including improved hydrophilicity and surface-free energy of the implant, chemical activation of the surface, reduced atmospheric contamination, and enhanced interactions with osteoblasts and proteins [10,11]. In fact, SLActive® has been shown to have 162% higher fibronectin adsorption compared to other surface types, including SLA [12]. Nonetheless, rough surfaces like those found in SLActive® can encourage biofilm development, raising the chances of peri-implantitis. This risk is particularly elevated in individuals with a history of periodontitis [13].

This case report illustrates that by adhering to appropriate surgical, prosthetic, and hygiene protocols, short (SLActive®) implants can effectively restore severely resorbed posterior alveolar ridges, even in individuals with a background of periodontal disease.

## Case presentation

**Figure 1:**
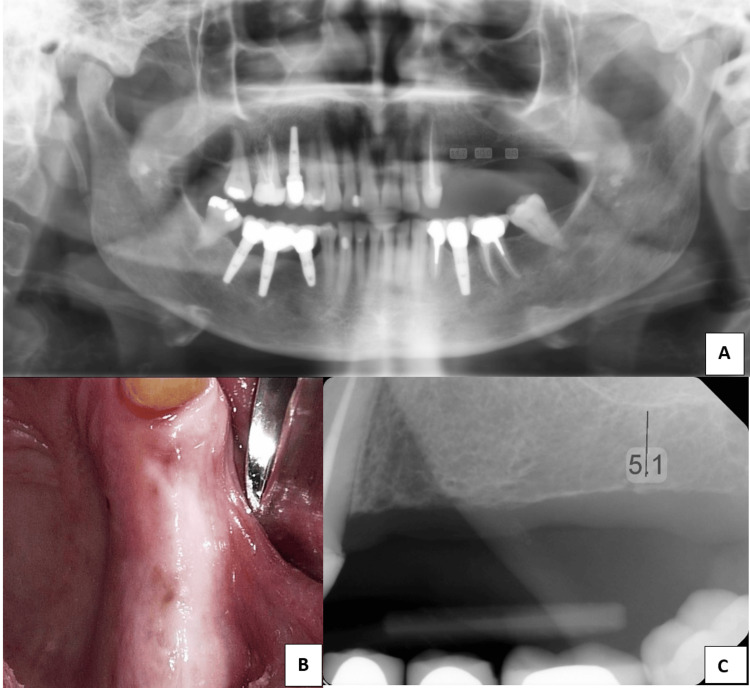
(A, B) Pre-surgical X-rays and clinical photograph. (C) The maxillary left ridge is severely resorbed, with noticeable pneumatization of the maxillary sinus.

**Figure 2:**
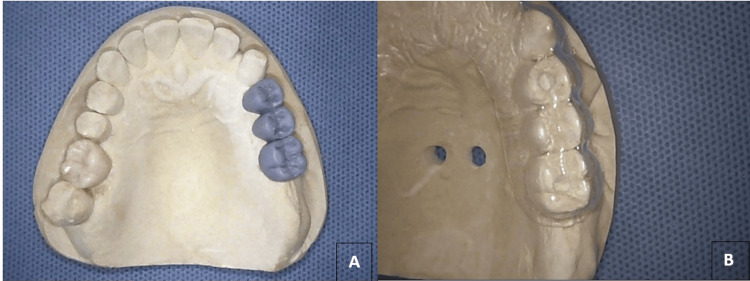
(A) Maxillary cast with a three-unit wax-up fixed partial denture. (B) Customized surgical stent for the maxillary arch, featuring two holes at the planned implant fixture osteotome locations (areas of #24 and #26).

In December 2018, a 67-year-old lady was referred to a periodontic clinic to replace her missing teeth with implants in areas of #24 and #26. The teeth loss was due to periodontal diseases (Figure 1). Her medical history did not reveal any significant issues. To determine the appropriate size, location, and type of implant for her treatment, orthopantomography (OPG) and periapical X-rays were taken (Figure 1). The patient already replaced the areas of #15, #35, #44, #45, and #46 with dental implants, so it was acceptable to deal with implant surgery. The X-ray examination revealed severe bone resorption in area #26, with left maxillary sinus pneumatization. The treatment options were explained to the patient, including sinus elevation with bone grafting or short implants. Treatment options were carefully explained with possible side effects and complications. The patient chose a short implant because of low morbidity and shorter treatment time. Alginate impression was taken to obtain diagnostic wax-up and surgical stent (Figure 2).

Prior to the surgery, the patient was instructed to rinse with a chlorhexidine mouthwash of 0.2% for two minutes prior to the procedure. Local anesthesia, containing lidocaine with adrenaline at a ratio of 1:100000, was administered using two carpules. The fit of the surgical stent was carefully checked before proceeding. The author then inserted implants in the #24 and #26 areas, utilizing a series of osteotomies with surgical drills. No bone grafting or sinus lifting was necessary, and the implants were placed with satisfactory primary stability. The primary stability was checked using percussion testing and insertion torque. Two implants with a titanium-zirconium chemically modified, SLA surface (Roxolid® SLActive®) were used. These were bone level, Regular CrossFit® (Straumann Group), with dimensions of 𝝓 4.1*8 mm and 𝝓 4.1*10 mm.

**Figure 3:**
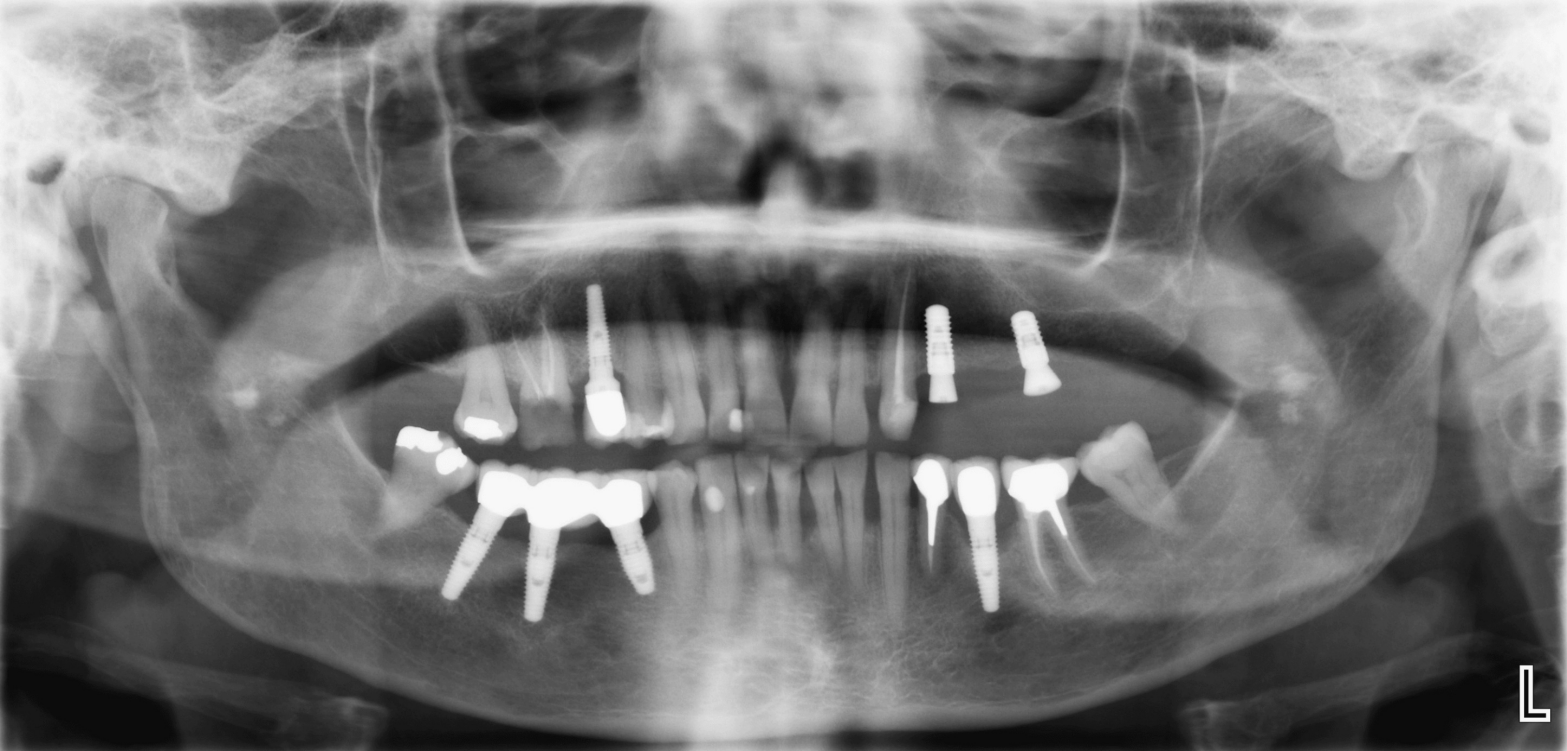
Two implant fixtures made of titanium-zirconium (Roxolid® SLActive®) were surgically placed in the areas of #24 and #26 (𝝓 4.1×10 mm and 𝝓 4.1×8 mm) and covered with screws. Later, the screws were replaced with healing abutments.

**Figure 4:**
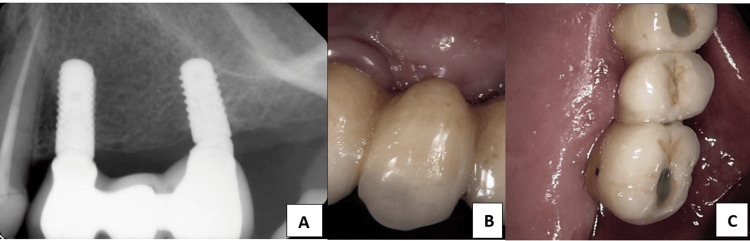
(A) Periapical X-ray showing the two implants restored and splinted together with a fixed partial denture (fixed prosthesis). (B, C) Clinical photos taken with an intraoral camera (Dentsply Sirona’s intraoral scanner (York, USA)) for the fixed partial denture.

After placing the cover screws, the flap margins were carefully approximated using interrupted sutures to ensure tension-free primary wound closure. A periapical X-ray was taken at the end of the surgery. Postsurgical medications were prescribed including analgesic and chlorohexidine mouthwash 0.12% for seven days. After four months, the implants were exposed and healing abutments were placed (Figure 3). Two weeks later, an impression was made to create a fixed cemented prosthesis. A porcelain-fused-to-metal fixed prosthesis (fixed partial denture) was cemented by temporary cement (Figures 4A-4C). Zinc oxide eugenol cement (Prime-Dent Temporary Cement®; Prime Dental Products, Chicago, USA) was used. The implant-supported fixed prosthesis was checked for occlusion after cementation and in every visit.

**Figure 5:**
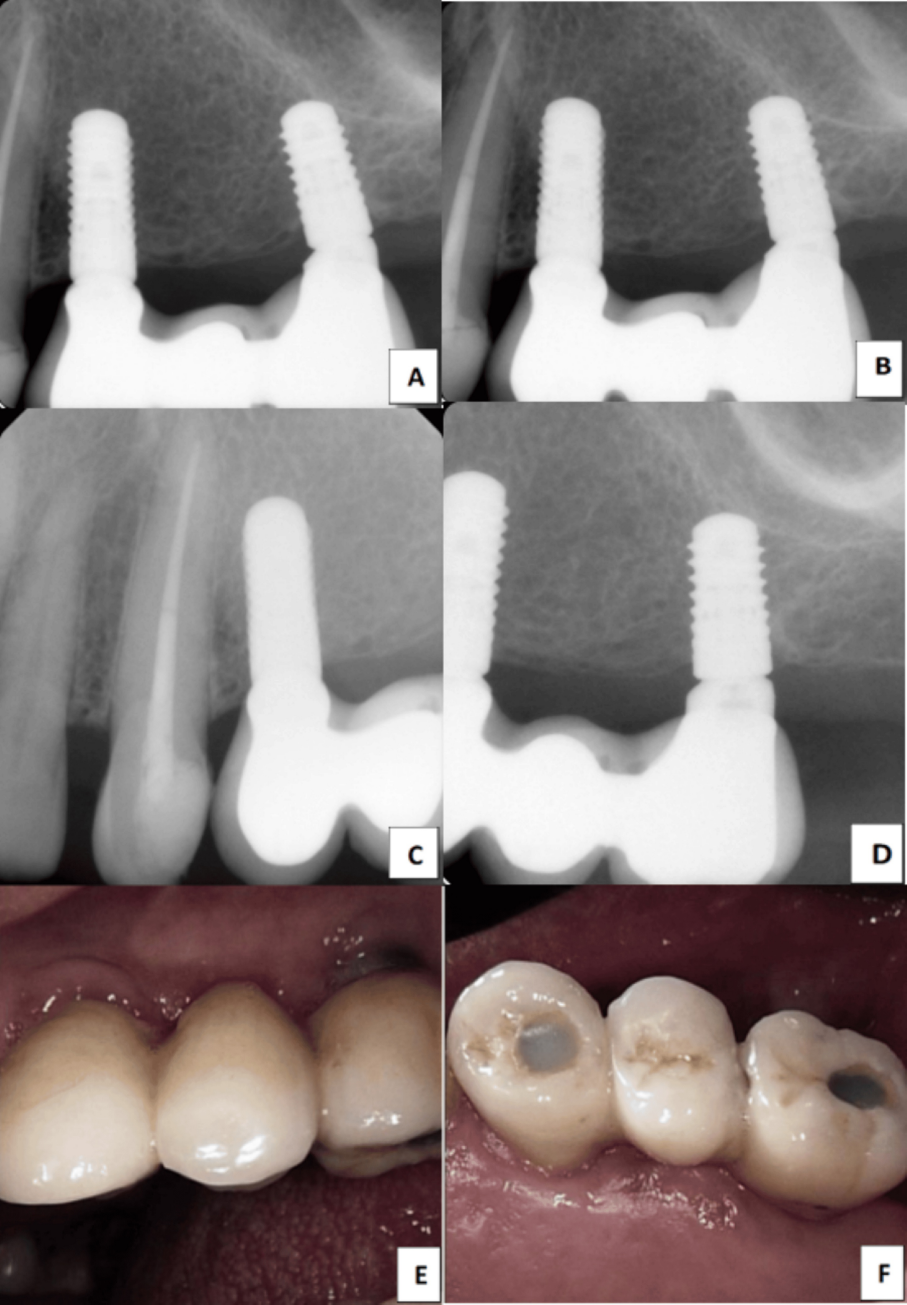
Follow-up images (A) The 2021 X-ray shows the two implants in the areas of #24 and #26. (B) The 2023 X-ray of the same implants. (C, D) The 2024 X-rays were taken for the #24 and #26 implants and prostheses. (E, F) Clinical photographs taken with an intra-oral camera in 2024, showing the same prosthesis after six years (E: Buccal view, F: Occlusal view).

The patient underwent a well-established oral hygiene protocol, which is typically used for all periodontal patients who are undergoing implant rehabilitation. The main goal of this protocol is to achieve and maintain optimal health of both hard and soft tissues. The protocol included regular instructions for oral hygiene, in-office dental hygiene, measurements of probing pocket depth (PPD), bleeding upon probing, gingival level, implant mobility, and prosthesis occlusion every four months. Additionally, radiographic follow-ups using intraoral periapical X-rays were done almost every 8-12 months. Throughout the follow-up period (six years), no signs of peri-implantitis, pathological PPD, or calculus were observed (Figure 5).

## Discussion

The biomechanical rationale for using short implants lies in the fact that the crestal region of the bone-implant interface bears the majority of the load from the prosthesis [14]. Consequently, short implants can be successfully utilized in regions with substantial resorption of the alveolar ridges, demonstrating success and survival rates comparable to those of longer implants placed alongside guided bone regeneration [15].

Short dental implants can be beneficial for patients with limited bone height, offering a less invasive option. However, they come with significant disadvantages, such as decreased initial stability and anchorage due to their reduced surface area, which can lead to increased implant mobility and a higher risk of failure. They are also more vulnerable to occlusal forces, potentially causing bone loss around the implant. Additionally, achieving optimal crown-to-implant ratios can be challenging, impacting both function and aesthetics. Thus, while short implants can be suitable in specific cases, careful patient selection and surgical technique are crucial for successful outcomes.

This case study aims to present a successful rehabilitation of a severe atrophic maxillary area that was affected by maxillary sinus pneumatization. The patient also had a history of periodontitis. The chosen treatment approach involved the use of short implants, splinted with standard-length implants, which have been found to have lower complication rates [16].

The study by Fan et al. [16] compared the rate of survival and complications of short implants (5-8 mm) to long implants (>8 mm) through a systematic review and meta-analysis of randomized clinical trials. The primary outcome measures were survival rate and complications, while secondary measures included costs and surgical time. The review found no significant difference in survival rates between the two groups, but short implants had lower rates of complications [16].

Following the healing period, a definitive prosthesis was created to splint the implants. Several finite element analyses have shown that the distribution of occlusal load on the splinted implants resulted in a decrease in the forces exerted on the implant bodies [17]. In addition, a study by Theoharidou et al. [18] demonstrated that splinted extra-short implants have statistically significantly lower rates of prosthetic complications and implant failures than non-splinted implants [18].

The case in this report demonstrates that proper surgical technique may contribute to the effective long-term success of short implants, including patients with periodontitis. A key factor in implant surgery is the strategic positioning within the bone structure. Specifically, it is vital that the entire surface area of the implant and a minimum of 0.5 mm of its smooth coronal region remain completely submerged in bone tissue; moreover, there should be a 1.5 mm width for both buccal and palatal crestal bones surrounding the implant site. This placement position has been supported by findings from Gil et al., which demonstrate that positioning implants 0.5 mm below bone level results in lower mechanical stress distribution compared to other placement positions. Such reduced stress levels are significant because they correlate with higher bone-to-implant contact (BIC) values observed in vivo after three to six weeks post-implantation - a crucial period for osseointegration. Furthermore, this sub-bone level placement encourages vertical bone growth up to the bone level, thereby enhancing stability and integration over time [19].

Roxolid® SLActive® surface implants offer substantial benefits in the field of dental implantology, marked by their enhanced osseointegration and biomechanical performance. The innovative combination of Roxolid®, a titanium-zirconium alloy, with the hydrophilic SLActive® surface treatment, creates a material that surpasses traditional titanium implants in both strength and reliability. Studies have indicated that Roxolid® implants can endure higher mechanical loads, which allows for reduced diameters without compromising stability [7,8]. This is particularly advantageous for patients with narrow jawbones or those requiring minimally invasive procedures. Additionally, the SLActive® surface is engineered to hasten early healing through its superior wettability and support of blood clot formation at the implant site [20]. Clinical trials have demonstrated improved BIC rates during critical early weeks post-surgery, resulting in faster overall treatment times and increased predictability in outcomes [7,8,20]. Consequently, these properties enable dental professionals to manage complex cases more effectively while simultaneously providing patients with quicker recovery periods and long-term success.

## Conclusions

Residual alveolar ridges often experience significant resorption following tooth extractions. In particular, the posterior region of the maxillary residual alveolar ridges is notably weakened and thinned due to the pneumatization of the maxillary sinus after teeth are lost. These conditions present considerable challenges for oral surgeons and periodontists who aim to place dental implants in these areas to restore missing teeth. Procedures intended to rehabilitate the atrophied maxillary ridges are typically complex, time-consuming, and costly. Additionally, these rehabilitation surgeries frequently require the use of autogenous or synthetic bone graft materials and involve intricate surgical techniques, which necessitate extra steps and careful management, contributing to higher morbidity rates.

In this case study, the author successfully rehabilitated the atrophied maxillary ridge in the posterior left region using two dental implants in a single, simplified surgical procedure. This approach eliminated the need for graft materials, maxillary sinus osteotomy, and other complex interventions. The surgical and prosthetic techniques employed in this case are detailed to address the challenge overcome and increase the survival rates of the dental implants, ultimately aiding the patient in restoring function and aesthetics in her maxillary dental arch. The patient presented with periodontitis, which increased the risk of implant loss due to peri-implantitis. However, with stringent maintenance follow-ups and exceptional oral hygiene, the implants thrived successfully in her mouth for six years without any complications.
